# Loss of cPLA2α function attenuates inflammation and epithelial thickening in a mouse model of *Haemophilus influenzae*-mediated COPD exacerbation

**DOI:** 10.1016/j.crmicr.2026.100556

**Published:** 2026-01-22

**Authors:** Brice Lagrange, Fatima Benmohamed, Alice Eon-Bertho, Laurette Malleret, Alexis Trecourt, Olivier Glehen, Lhousseine Touqui, Abderrazzak Bentaher

**Affiliations:** a Equipe, Inflammation et Immunité de l’Epithélium Respiratoire, INSERM, France; b Sorbonne Université, INSERM UMR-S938, Centre de Recherche Saint-Antoine (CRSA), Paris, France; c Mucoviscidose and Bronchopathies Chroniques, Département Santé Globale, Institut Pasteur, Paris, France; d UR 3738 CICLY, Faculté de Médecine Lyon Sud, France

**Keywords:** Haemophilus influenzae, COPD exacerbation, Cytosolic phospholipase 2α, lung inflammation, Lung injury

## Abstract

Among the characteristics of chronic obstructive pulmonary diseases (COPD) especially emphysema are chronic airway inflammation and tissue destruction in response to the primary etiologic factor, cigarette smoking (CS). Importantly, COPD is aggravated by respiratory infections. Approximately half of these exacerbations are caused by bacterial infections with *Haemophilus influenzae* (*H. influenzae*) being the most common pathogen. Unfortunately, the underlying mechanism(s) of these exacerbations remain poorly understood, resulting in scarce and ineffective therapies. In this study, we investigated the relative contribution of host cytosolic phospholipase 2α (cPLA2α) to this clinical manifestation. We established a mouse model that closely mimics COPD exacerbation in which cPLA2α‐deficient mice (cPLA2α‐KO) and their wild-type littermates were exposed to CS for 4 months intercalated with periodic intranasal infections with *H. influenzae*. Our findings revealed an increased expression of a series of inflammatory mediators in the lungs of wild type, but not cPLA2α‐KO, mice. Furthermore, unlike cPLA2α‐KO lungs, wild type lungs exhibited a markedly thickenned airway epithelium. Our work shed light on a novel mechanism of COPD exacerbation that involves cPLA2α.

## Highlights

• Development of mouse model that closely mimics Haemophilus influenzae-mediated COPD exacerbation.
• Gene targeting approach reveals that cytosolic phospholipase 2-a (cPLA2-a) alters lung inflammatory response using COPD exacerbation experimental model.
• Also, cPLA2-a contributes to airway epithelium thickening in this clinical manifestation.
• Novel mechanism of bacterial infection-mediated COPD exacerbation involving cPLA2a.

## Graphical abstract

**Figure:**
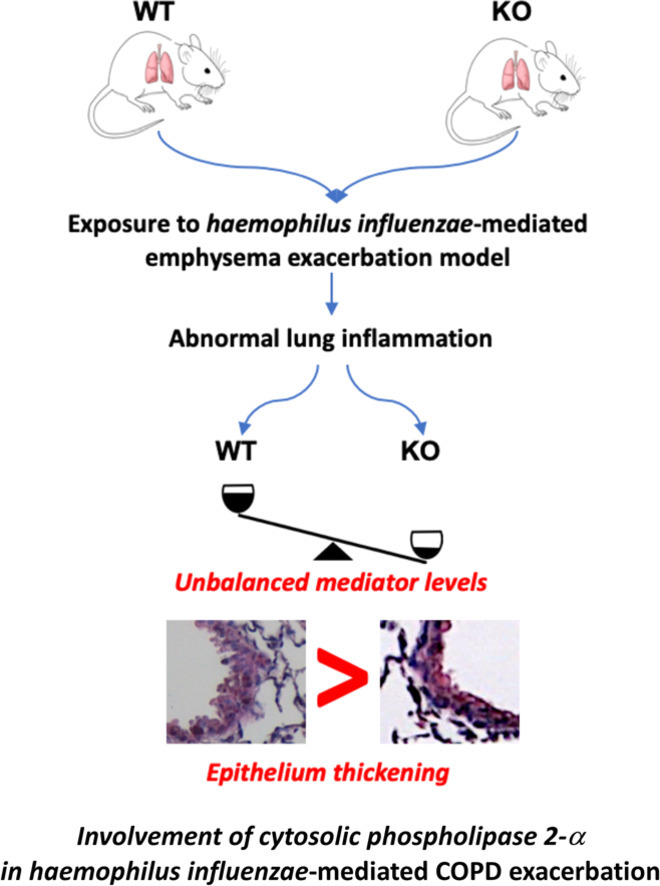

## Introduction

Chronic obstructive pulmonary diseases (COPD) with emphysema regarded as a major component of COPD are characterized by chronic airway inflammation and progressive tissue destruction primarily driven by cigarette smoke (CS) exposure. This results in airflow limitation that is incompletely reversible (Celli et al., 2015; Croxton et al., 2003). Importantly, exacerbations of COPD (on average two to three times per year) by various environmental factors contribute considerably to the severity of this pathology (Bhowmik et al., 2000). Of note, COPD with or without exacerbations typically manifests in mid to late adulthood and remains a major global health burden, currently ranking as the fourth leading cause of death worldwide ((Chronic obstructive pulmonary disease (COPD) 2025; Wedzicha and Seemungal, 2007)).

Approximately half of exacerbations are precipitated by bacterial infections, with *Haemophilus influenzae (H. influenzae)* being the most commonly isolated pathogen (Sethi et al., 2002; Suissa et al., 2012). These infection-mediated exacerbations are widely recognized as an important cause of overwhelming lung inflammation leading to hospital admissions, morbidity, and mortality. Unfortunately, despite their clinical impact the mechanisms governing the inflammatory amplification during COPD exacerbations remain poorly understood resulting in scarce and ineffective therapies.

Cytosolic phospholipase 2α (cPLA2α) belongs to a family of enzymes that hydrolyze membrane phospholipids leading to the formation of lysophospholipids and the concomitant release of arachidonic acid (AA) (Six and Dennis, 2000). The latter is further converted by cyclooxygenase (COX) and lipoxygenase (LOX) into pro-inflammatory mediators, such as prostaglandin E2 (PGE2) and leukotriene B4 (LTB4), respectively (Peters-Golden et al., 2005). These metabolites are known to regulate neutrophilic inflammation, host defense responses, and tissue injury. These pathways are highly relevant to the inflammatory milieu characteristic of infection-driven COPD exacerbations. cPLA2α has been shown to contribute to the pathogenesis of several inflammatory lung diseases including asthma, cystic fibrosis and lung fibrosis (Kita et al., 2006; Leslie, 2015; Dif et al., 2010). While the role of various PLA2 enzymes in COPD has been reported in either human or animal studies (Yu et al., 2024; Zhang et al., 2025), the specific contribution of cPLA2α to the development and severity of COPD exacerbations particularly those triggered by bacterial infection has not been addressed. Given its established role in lipid mediator biosynthesis and inflammatory amplification, we hypothesized that cPLA2α may critically modulate host inflammatory responses during COPD exacerbations.

To test this hypothesis, we developed a mouse model that closely recapitulates key features human COPD exacerbation, in which mice deficient in cPLA2α were exposed to cigarette smoke for 4 months intercalated with episodic intranasal infections with non-typeable H. influenzae (NTHi). Our findings identify for the first time cPLA2α as a pathogenic contributor to COPD exacerbations since its genetic ablation attenuated NTHi-driven exacerbation responses, at least in mice.

## Materials and methods

### Reagents, cells, bacteria and mice

Purified ETNA elastin was obtained from Elastin Products Company (Owensville, MO, USA). All tissue culture reagents and other chemicals were reagent grade and were purchased from Invitrogen or Sigma-Aldrich, unless otherwise stated.

The mouse macrophage-like cell line Raw 264.7 were obtained from ATCC (Manassas, USA). The MLE15 cell line was a gift from J whitestt, Cincinnati Children's Hospital, USA). Cells were cultured in Dulbecco’s modified Eagle medium (DMEM) with 10 % fetal bovine serum (FBS) and 1 % penicillin/streptomycin at 37 °C in 5 % CO_2_ as previously described (Raymond et al., 2009; Bouyssi et al., 2023). It must be emphasized that in this study, we focused on macrophages and epithelial cells because they are considered as sentinel cells that play critical roles in the initiation and progression of COPD and its exacerbation. However, this does not preclude the relative contribution of other cell types such as endothelial cells in disease pathogenesis (Khan and Ilies, 2023; Leslie, 2015).

In this work, we used *non-typeable Haemophilus influenza (NTHi)* strain 11P6 (kindly provided by M. S. Sethi, University of Buffalo, Buffalo, New York) (Fernaays et al., 2006). This strain was isolated from a COPD patient with acute exacerbation and subsequently confirmed as the causative agent of the exacerbation (Gaschler et al., 2009). Overnight cultures of single colonies were grown in 10 ml brain-heart infusion (BHI) broth supplemented with hemin and nicotinamide adenine dinucleotide (NAD) (SIGMA-Aldrich) at 37 °C until the late exponential phase (3 h) (Gaschler et al., 2009). Bacteria were collected by centrifugation (500 x g, 10 min), washed twice, and resuspended in 1 ml phosphate-buffered saline (pH 7.4). The optical density (OD) of the bacterial culture was determined at 600 nm (OD 1 ≈ 10^9^ bacteria/ml). cPLA2α-deficient mice (cPLA2α-KO) were generated by targeted mutagenesis as previously described (Uozumi et al., 1997). Since cPLA2α-KO females do not undergo parturition, heterozygous cPLA2α−/+ females were mated with cPLA2α-KO males. All offspring were genotyped by PCR on tail genomic DNA using specific oligonucleotide primers and the resulting cpla2α -/- and WT babies were selected. The cPLA2α-KO and wild-type (WT) littermates mice (C57Bl/6 J, 8–10 weeks old) were housed in a pathogen-free facility and given food and water ad libitum.

Animal handling and procedures were approved by the Animal Studies Committee of our institution (Health and Animal Protection Office, Authorisation # Lyon Sud 2012–003) in accordance with the European Union Directive 2010/63/EU on the protection of animals used for scientific purposes.

### NTHi intranasal infection and bacterial clearance

Groups of both cPLA2α-KOand WT mice (*n* = 10/genotype divided equally between females and males) were intranasally (i.n.) infected with *NTHi* (10^6^ CFUs) and monitored over time (Boxio et al., 2016). Briefly, mice were anesthetized by intraperitoneal injection of 50 mg/kg ketamine hydrochloride and 5 mg/kg xylazine hydrochloride. Mice were then challenged intranasally with 50 μl of saline buffer (PBS) containing a predetermined dose of bacteria. Control mice (*n* = 8 mice/genotype) were challenged with 50 μl of sterile PBS alone. WT and cPLA2α-KO mice were monitored daily for up to one week. One WT mouse and one cPLA2α-/-mouse died during intranasal instillation and one cPLA2α-/-mouse was lethargic for the first two days but recovered thereafter.

In separate experiments, groups of cPLA2α-KO and WT mice (*n* = 10/genotype) were *i.n.* infected with *NTHi*. At 24 h post-challenge, half of each group was euthanized and their BAL fluids were collected with 1 ml PBS, pH 7.4 that was cycled in three times and total cell counts were performed using a hemacytometer. The remaining halves were euthanized at 96 h post-infection and collected BALs were plated on chocolate agar supplemented with NAD (V factor) and hemin (X factor) to assess bacterial clearance (CFUs) as previously described (Guyot et al., 2014).

### Mouse NTHi-mediated COPD exacerbation

To establish the mouse model mimicking *NTHi*-mediated COPD exacerbation, we performed the same experimental design as previously reported (Guyot et al., 2014; Bouyssi et al., 2025) with the following modifications: groups of WT and cPLA2α-/- mice were exposed to 2 (3R4F) cigarettes a day for 5days a week for 4 months. Mice were intranasally (i.n.) challenged with a predetermined dose of 10^6^ CFUs of *NTHi* at the end of the second and third months. There were various groups of mice (*n* = 10 divided equally into females and males): group exposed to filtered air only with two intranasal instillations of PBS (controls), mice exposed to cigarette smoke (CS) only, mice challenged i.n. with *NTHi* only, and mice exposed to CS and i.n. challenged with *NTHi*. Mice were briefly anesthetized with 3 % isoflurane before bacterial instillation. Mice were sacrificed one month after the last instillation and processed as described below. No deaths were observed in mice following bacterial infection.

### Processing of COPD exacerbation mice

Mice euthanized by cervical dislocation and the lungs were processed as previously described (Guyot et al., 2014).

### Bronchoalveolar lavage processing

The lungs were lavaged in situ (bronchoalveolar lavage, BAL) with 1 ml PBS, pH 7.4, and cycled in three times. Identical recoveries of BAL (700 µl/mouse) were obtained for each mouse.

Total cell counts and differential counts were immediately performed on aliquots of BAL fluids. Total cell counts from BAL fluids were performed by a hemacytometer. For differential counts, cells were cytospun, Wright-stained, and identified using standard morphological criteria.

Cell-free BALs were prepared as follows: for each experimental condition and in each group, three pools of an equal volume of cell-free BALs (two pools of three samples and one pool of four samples) were prepared, aliquoted and stored at -80 °C until use.

### Lung tissue processing and staining

The lungs were rinsed, and the right halves were processed for histology. The left halves were snap-frozen in liquid nitrogen for protein or total RNA extraction.

Right lung halves. For histology analyses, right lung halves were inflated in situ with 10 % formalin and fixed for 24 h, embedded in paraffin and serial five µm-thick tissue sections were prepared. Tissue section slides were hematoxylin and eosin (H&E)-stained according to the manufacturer's recommendations (CliniSciences, Nanterre, France) and examined by light microscopy.

Left lung halves. For host inflammatory response analyses, in each group and per experimental condition and, three pools of equal RNA amounts were prepared per genotype (two pools of three RNA aliquots and one pool of four RNA aliquots) for RNA analyses. All the samples were aliquoted and stored at -80 °C until use.

### Morphometry

Pulmonary tissue destruction was assessed by morphometric analyses of lung histological changes and determining the average interalveolar distance. This latter was estimated by calculating the mean linear intercept (L_m_), which is an estimate of the average wall-to-wall distance of the alveoli (Guyot et al., 2014). Briefly, for each mouse, ten randomly digitized images of representative H&E stained left lung tissues were captured in a blinded fashion (A.B. or L.M.) using a DM750 microscope coupled to a digital camera module ICC50 and analyzed using Image J 1.33 image analysis software (http://rsb.info.nih.gov/ij/). Airspace enlargement was quantified by measuring L_m_. Bronchial wall thickness was assessed using Image J 1.33 software.

### Cytokine antibody arrays

Cell-free BAL pools were processed to assess the levels of various cytokines as previously described (Benabid et al., 2012) using the RayBio mouse cytokine antibody arrays C3 and 4 (RayBiotech, Tebu-Bio, Le Perray-en-Yvelines, France). Briefly, equal volumes of cell-free BAL fluids (1000 μL) were added to antibody-coated membranes and detection of immunoreactive cytokines was performed after sequential incubation of the membranes with biotinylated anti-cytokine antibodies and streptavidin-horseradish peroxidase and visualization by enhanced chemiluminescence. Images were obtained using a ChemiDoc XRS imaging system (Bio-Rad Laboratories). Semiquantitative analysis by densitometry was performed on captured images using Quantity One 1-D analysis software version 4.5.2 (Bio-Rad Laboratories). Spots of interest were normalized to an internal control after subtraction of a representative background sample. Cytokine antibody array assays were performed on all BAL fluid pools.

### Preparation of cigarette smoke extract (CSE)

CSE was freshly prepared as previously reported (Bouyssi et al., 2023). Smoke from two cigarettes was bubbled into 1 mL of PBS. This solution was considered to be 100 % CSE. The extract was filter sterilized (0.22 μm filter) and used immediately.

### Cultured cells studies

To complement the in vivo findings and to determine whether the inflammatory responses observed in the mouse model could originate directly from macrophages or airway epithelial cells, we performed in vitro experiments using two murine cell lines relevant to lung pathophysiology. As mentioned above, we focused on macrophages and epithelial cells because they are considered as sentinel cells that play critical roles in the development of COPD and its exacerbation (Tetley, 2002; Holtzman et al., 2014).

Both Raw 264.7 and MLE15 cells (10^6^ cells/ml) were grown to subconfluence in 24-well plates as described above. Cells were then incubated alone or in the presence of CSE at 5 % (v/v) for 1 h and then with DMEM and NTHi at a multiplicity of infection (MOI) of 10 for 1 h as recently reported (Bouyssi et al., 2023). Cells were scraped and total RNA was extracted and quantified. RNA aliquots were then prepared at defined concentrations and stored at -80 °C until use.

### Enzyme immunoassay (EIA)

Mouse cell-free WT and cPLA2α-KO BAL fluids were processed to assess the levels of mediators of interest by ELISA according to the manufacturer’s instructions (R&D systems (Minneapolis, USA) (Filgueiras and Possmayer, 1987). LTB4 levels were analyzed using EIA kits according to the manufacturer's protocols (Cayman Chemicals, France). Briefly, equal volumes of supernatants or cell-free BAL fluids (100 μl) were added to antibody-coated plates and detection of immunoreactive cytokines was performed.

### Measurements of cPLA2α activity

Lung tissues or cell culture pellets were lysed according to Filgueiras and Possmayer (Filgueiras and Possmayer, 1987) and centrifuged for 5 min at 1000 xg to remove debris. Protein concentrations were determined using a kit from Pierce (Thermo Scientific, Rockford, IL, USA). Extracts with equal amounts were incubated for 30 min with 1 mL vesicles containing 6 nmoles of 1-palmitoyl-2[14C]arachidonoyl-sn‑glycero-3-phosphorylcholine (>53 mCi·mmol−1) (Perkin-Elmer, Boston, MA, USA) and 4 nmoles of diacylglycerol (Sigma), in the presence of 5 mM CaCl2 and 1 mM 2-mercaptoethanol. This assay selectively detects cPLA2α activity because iPLA2 (calcium independent phospholipase A2) activity does not require calcium, and 2-mercaptoethanol inhibits sPLA2 (secretory phospholipase A2) but not cPLA2α activity (Hidi et al., 1993).

### Reverse transcription and real-time PCR

Total RNA isolation from cultured cells or left lung tissues was performed using the MasterPure™RNA Purification Kit according to the manufacturer’s protocol (Epicentre, Biotechnologies). RNA samples (1 μg) were reverse-transcribed and cDNAs were used as templates for qPCR using TaqMan® probes (Thermo-Fisher). Amplification was carried out on an AriaMx Real-Time PCR thermocycler (Agilent, Vénissieux, France), using the 2^-ΔΔCt^ method (Bouyssi et al., 2025) to determine the relative expression of genes of interest. The gene coding for hypoxanthine-guanine phosphoribosyltransferase (HPRT) was used as housekeeping gene.

Mouse qPCR primers:

OPN

Forward: 5′-TGG AAC ATC AGA GCC ACA AG-3

Reverse: 5′-TCG GAA TTT CAG ATA CCT ATC T-3′

MIP-2

Forward: 5′-GTG AAC TGC GCT GTC AAT GC-3′

Reverse: 5′-ACT CAA GCT CTG GAT GTT CTT GAA-3′

TGF-Beta

Forward: 5′-CTC CCG TGG CTT CTA GTG C-3′

Reverse: 5′-GCC TTA GTT TGG ACA GGA TCT G-3′

Vimentin

Forward: 5′-CCA ACC TTT TCT TCC CTG AA-3′

Reverse: 5′-TGT TCT TTT TGA GTG GGT GTC A-3′

HPRT

Forward: 5′-AGC CTA AGA TGA GCG CAA GT-3′

Reverse: 5′-TTA CTA GGC AGA TGG CCA CA-3′

### Statistical analyses

Statistical tests were performed using Prism version 8.4.2 software (GraphPad Software, La Jolla, CA, US) and data were expressed as mean and standard deviation (SD). Statistical tests were used to investigate differences in ΔCt for RT-qPCR and concentrations (pg/mL) for ELISA. To compare the differences between groups, normality was verified using the Shapiro-Wilk test and then, either ordinary one-way ANOVA with Tukey’s multiple comparisons test, Kruskal-Wallis’ test with Dunn’s multiple comparisons test or Brown-Forsythe and Welch’s ANOVA with Dunnett’s multiple comparisons test were performed. Differences were considered significant at *p* ≤ 0.05.

## Results

### Deficiency in cPLA2α does not protect mouse lungs from alveolar space damage in the COPD exacerbation model

**Fig. 1:**
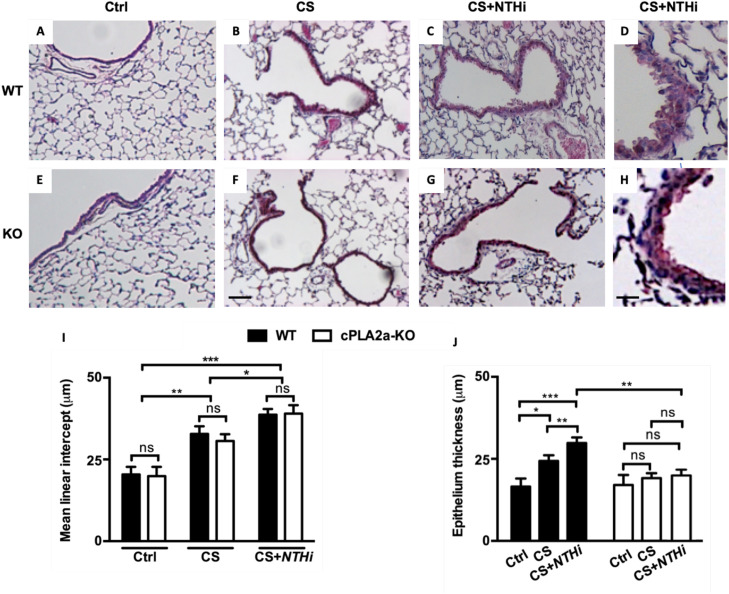
**Histological analyses of lung damage in mice after chronic exposure to CS ± *NTHi* infection.** Deficiency of cPLA2α does not protect mice against lung tissue destruction after long-term exposure to CS. Representative micrographs of lung tissue from unexposed (Ctrl; **A** and **E**), CS-exposed (**B** and **F**), and CS-exposed**+***NTHi* (**C** and **G**) mice. Both mouse genotypes exhibited marked alveolar enlargement in response to CS or CS*+NTHi*. **D and H/** insets of **C** and **G** respectively**. I/** No statistically significant differences of Lm between WT lungs (black bars) and cPLA2α-KO lungs (white bars) (**CS and CS*+NTHi***). Exposed WT and cPLA2α-KO mice had much higher Lm values than unexposed mice (**Ctrl** lanes). **C,D and G,H /** Unlike cPLA2α-KO (**G,H**), WT mice (**C,D**) showed marked thickening of the airway epithelium in response to CS+NTHi, an observation that was confirmed by morphometric analyses (**J**). Scale bars: 150 μm. Data are expressed as means ± SEM and are representative of three independent experiments. *** *p* < 0001; ** *p* < 0,01, * *p* < 0,05; ns, not significant.

The overall lung histology of age-matched sham and non-smoking WT and cPLA2α-KO mice appeared normal (Fig. 1A,E). However, compared to unchallenged mice, exposure of both mouse types to CS alone for 4 months resulted in an increase but similar of both enlargement of alveolar spaces and disruption of alveolar walls (Fig. 1B,F) that was further amplified when CS exposure was intercalated with *NTHi* i.n. instillations (Fig. 1C,G). Indeed, calculations of L_m_ confirmed these observations. When compared control lungs, the L_m_ of both CS-exposed cPLA2α-KO and WT lungs increased but were similar, (Fig. 1I, CS). These Lm further increased following *NTHi*-mediated exacerbation (Fig. 1I, CS+*NTHi*). Therefore, cPLA2α deficiency does not contribute to lung protection against tissue destruction in either CS-induced COPD or *NTHi-*exacerbated COPD models. Of relevance, prior to carry out experiemnts with the COPD exacerbation model, we ensured that bacterial clearance of *H. influenzae* by mice was not impaired knowing that *H. influenzae* is a human-restricted pathogen. Our results confirmed that regardless mouse genotype, infection-induced abnormal inflammatory response had no bearing on host survival, the response to NTHi was predominated by neutrophils and bacterial clearance was similar between mouse groupe (**Supplementary figure 1**).

### Deficiency in cPLA2α prevents airway epithelium thickening in COPD exacerbation model

Focusing on the exacerbation experiment, in addition to the increased alveolar space enlargement, histological lung analyses revealed a much more thickened airway epithelia in WT mice compared to cPLA2α-KO mice following CS exposure intercalated wit *NTHi* lung infection (Fig. 1C,Fig. 1, Fig. 1D,H). This was further confirmed by morphometric analyses (Fig. 1J). Altogether, these findings suggest that uncontrolled cPLA2α activity promotes airway epithelial thickening in the setting of *NTHi*-mediated COPD exacerbation. All subsequent findings were focused on the role of cPLA2α on this latter experimental condition.

***cPLA2α is not required for lung recruitment of inflammatory cells.***

**Fig. 2:**
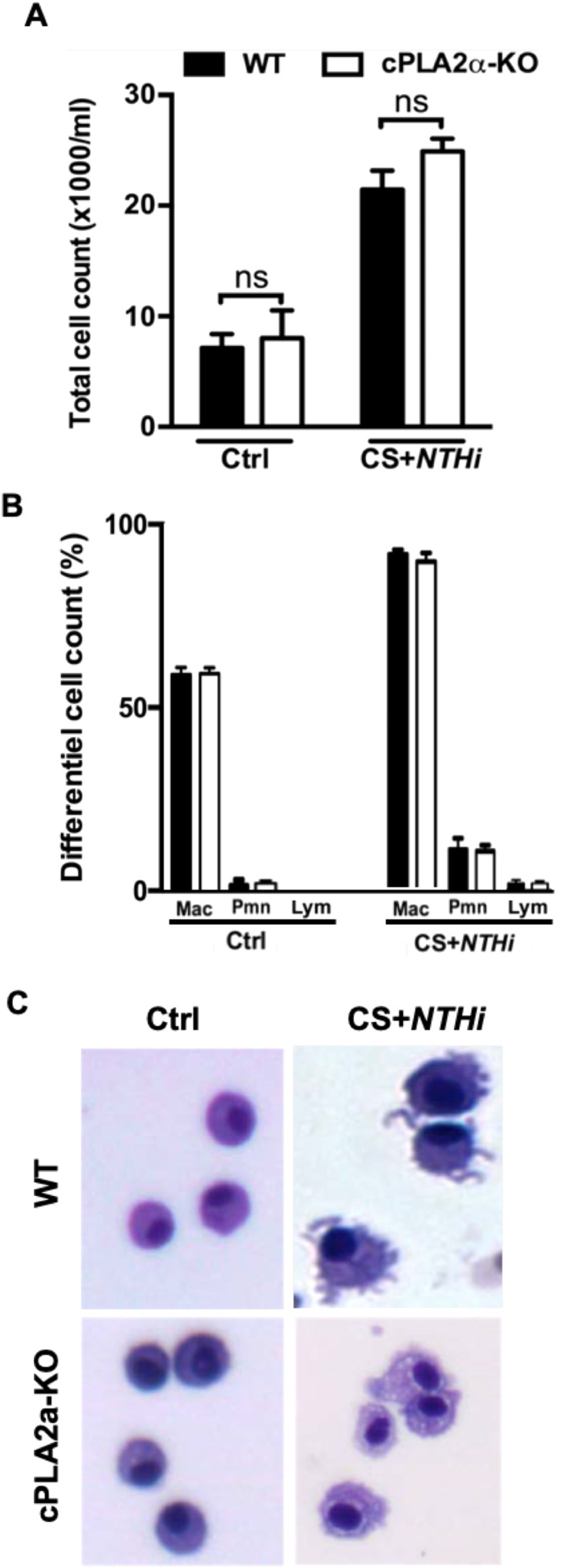
. **cPLA2α is not required for lung recruitment of inflammatory cells after chronic exposure to CS + *NTHi* infection. A/** Total cell counts were higher in BAL fluids of CS+NTHi-treated mice compared to control mice (Ctrl) but there was no difference in cell recruitment between cPLA2**α**-KO **(**white bars**)** and WT **(**Black bars**)** mice. **B/** Differential cell counts were comparable in BAL fluids of control and CS+NTHi-treated mice. No significant differences were observed in the types of recruited cells, macrophages (Mac), neutrophils (Pmn) and lymphocytes (Lym) in BALs of control and CS+NTHi-treated mice. Note that macrophages are the predominant cells, followed by neutrophils. Data are presented as means ± SEM and are representative of three independent experiments. ns, not significant.

There was no difference in total cell recruitment between the WT and cPLA2α-KO in the COPD exacerbation model (Fig. 2A). Also, differential analysis of BAL fluids showed similar increase of total cell counts in both WT and cPLA2α-KO lungs, with macrophages being the predominant cells (Fig. 2B). As excepted, neutrophil and lymphocyte numbers increased significantly, but remained lower than macrophage numbers (Fig. 2B). These findings suggest that cPLA2α is not required in inflammatory cell migration in our model.

Because of its ability to process AA and generate downstream chemotactic mediators such as LTB4, cPLA2α could pave the way for immune cell migration into inflamed tissues. In vivo studies of our chronic inflammatory model showed active cPLA2α (**Supplementary figure 2A**). However, no statistically significant difference in LTB4 levels between both mouse genotypes (**Supplementary figure 2B**), hence contributing to the observed similar inflammatory cell recruitment in the presence or absence of cPLA2α. These findings suggest that the enzyme is not dispensable for inflammatory cell recruitment in this chronic lung inflammation model. But, when we subjected both types of mice to an acute inflammation model namely intranasal infection with *NTHi* and checked the levels of AA-derived metabolite LTB4 twelve hours later, we found a marked decrease of this metabolite in the absence of cPLA2α (**Supplementary figure 2C**).

***Decreased levels of inflammatory mediators in the COPD exacerbation model in the absence of cPLA2α.***

To obtain a broader overview of inflammatory mediator expression during COPD exacerbation, we performed cytokine antibody array profiling on pooled, cell-free BAL fluids from WT and cPLA2α-KO mice of our experimental model (Supplementary Figures 2 and 3). Our analysis revealed genotype-dependent differences in the relative abundance of multiple inflammatory mediators, consistent with an altered inflammatory milieu in the absence of cPLA2α. Because BAL samples were pooled per genotype, the array data are presented descriptively to illustrate global trends rather than to support quantitative comparisons or statistical inference. Importantly, these profiles complement the targeted, quantitative analyses shown in the main figures and further support the conclusion that cPLA2α contributes to shaping inflammatory responses during COPD exacerbation.

**Fig. 3:**
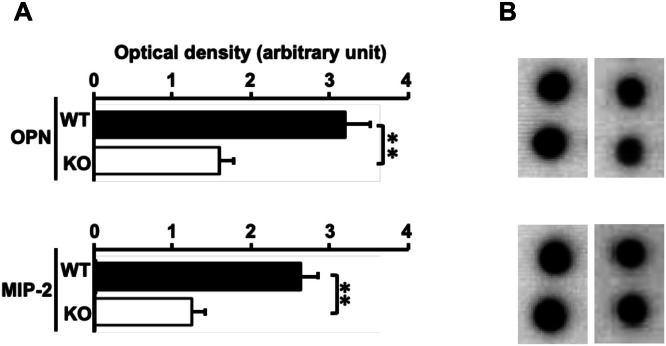
**Protein levels of OPN and MIP-2 in cell-free BAL fluids of challenged mice.** Shown are data of the representative mediators, OPN and MIP-2. **A/** Histograms of individual mediators, OPN and MIP-2, following densitometric analysis of cytokine antibody arrays of WT and cPLA2α-KO cell-free BAL fluids derived from COPD exacerbation model. Of note, level values of the mediators corresponding to unchallenged BAL fluids were subtracted from those of CS+NTHi-exposed BAL fluids. Data represent the mean ± SEM of three pool values. **, *p* < 0.01 for differences between genotypes. **B/** representative spots of OPN and MIP-2 on cytokine antibody array blots reflecting decreased protein levels in cell-free BAL fluids in the absence of cPLA2α. Left up and down panels and right up and down panels correspond to spots of WT and cPLA2α-KO cell-free BAL fluids derived from COPD exacerbation model, respectively. The cytokines are represented by standardized abbreviations. WT, wild type, and KO, cPLA2α-deficient. .

OPN and MIP-2 were then selected for further analysis based on their documented involvement in airway inflammation and COPD-related processes, together with their modulation in the BAL cytokine profiling data of WT versus cPLA2α-deficient mice **(Supplementary Figures 3 and 4)**. This focused analysis was intended to explore representative inflammatory mediators rather than to provide an exhaustive assessment of all altered cytokines. Also, it should provide relevant insight into downstream inflammatory pathways regulated by cPLA2α during COPD exacerbation. Semiquantitative analysis by densitometry found a marked increase of the proteins in cell-free WT BAL fluids when compared with those of cPLA2α -KO BAL fluids (Fig. 3). Of note, cytokine levels in cell-free BALs of untreated mice were insignificant regardless of mouse genotype and their values were subtracted from their corresponding cell-free BAL fluids of challenged mice.

### Decreased mRNA expression of OPN and MIP-2 in the COPD exacerbation model

**Fig. 4:**
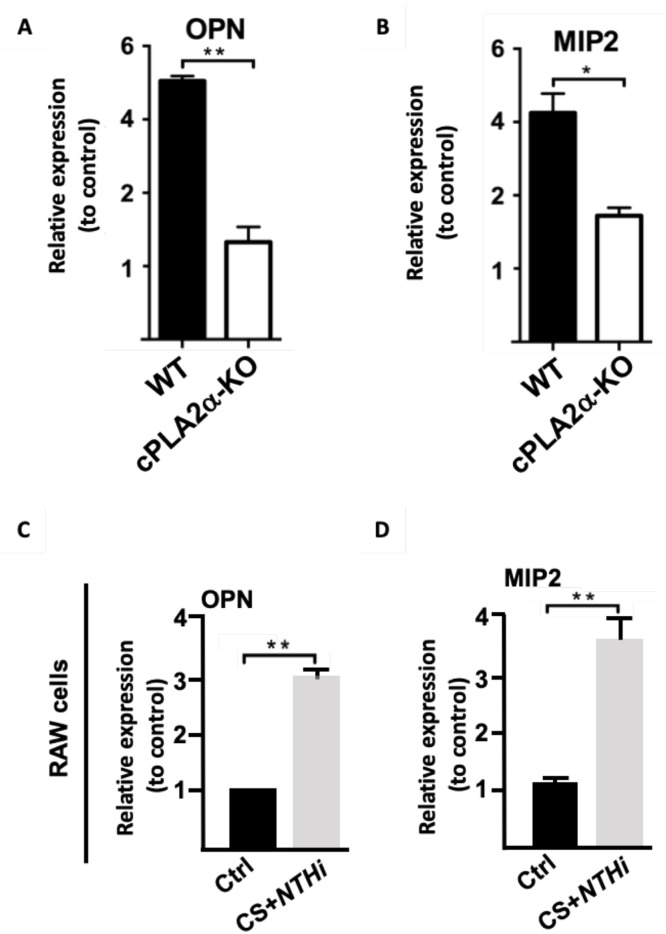
**Decreased lung OPN and MIP-2 mRNA transcript levels in the absence of cPLA2α after chronic exposure to CS + *NTHi* infection. A,B/** Results are expressed as fold increase of transcripts in CS+NTHi-exposed lungs when compared to unexposed lungs. Data shown correspond to one pool of equal amounts of RNA from CS+*NTHi*-exposed WT or cPLA2α-KO lung tissue homogenates. Similar data were obtained in the other two pools of each genotype. Data represent the means ± SEM. *p<0.05, **p<0.01. **Induced gene expression of OPN and MIP-2 in cultured cells in response to CS+NTHi treatment.** Subconfluent RAW-274.6 cells were incubated alone or in the presence of defined concentrations of CSE followed by a defined bacterial load of *NTHi* for designated periods of time . Changes in gene expression were analyzed by real-time RT-PCR using equal amounts of RNA from untreated and CS+NTHi-treated cells and normalized to GAPDH. **C,D/** Shown are representative histograms corresponding to the expression data of OPN and MIP2. Data represent the mean ± SEM of three values. *p<0.05, **p<0.01. Similar data were obtained in two replicate experiments. .

We hypothesized that the decreased levels of OPN and MIP-2 in cell-free cPLA2α-KO BALs could be attributed to changes in transcript expression of these mediators involving cPLA2α. To test the latter hypothesis, mRNA expression of OPN and MIP-2 was examined by real-time RT-PCR. As shown in Fig. 4A and B, levels of mRNAs encoding for OPN and MIP-2 were lower in cPLA2α-KO lungs compared to WT lungs in our experimental COPD exacerbation model. Of note, detected levels of OPN and MIP-2 transcripts in unexposed WT and cPLA2α-KO mice were insignificant and were subtracted from those of exposed samples for densitometric analyses. Taken together, these data strongly suggest a role for cPLA2α in inducing the expression of OPN and MIP-2 (confirming the cytokine antibody data), which contributes to the *NTHi*-mediated exacerbated inflammation.

Next, to begin understand the contribution of cPLA2α in the induction of this inflammatory response, transcript expression of mediators of interest was examined in cell culture studies using relevant murine cell lines. Of note, mouse lung comprises multiple cell populations that contribute differently to the inflammatory response during COPD exacerbations. Our in vivo data demonstrate that CSE + NTHi action amplifies airway inflammation, but do not identify which cell compartments initiate or sustain this response. To address this, we selected RAW264.7 macrophages. This cell line represents a standard cell model of to investigate lung macrophage responses to various insulting agents including bacteria and inflammatory stimuli. Resident macrophages are in fact, along with epithelial cells, the first point of contact for inhaled pathogenic factors ( e.g., cigarette smoke and bacteria) and principal source of cPLA2α-dependent eicosanoids.

First, we ensured that cPLA2α is active following exposure of the cells to CSE+NTHi (**Supplementary figure 4**). Next, we observed that such activity coincides with a significant increase in MIP2 and OPN mRNA transcripts when compared to untreated cells (Fig. 4C and B). These data support our in vivo findings.

***Decreased mRNA expression of TGF-β and vimentin in the COPD exacerbation model***.

**Fig. 5:**
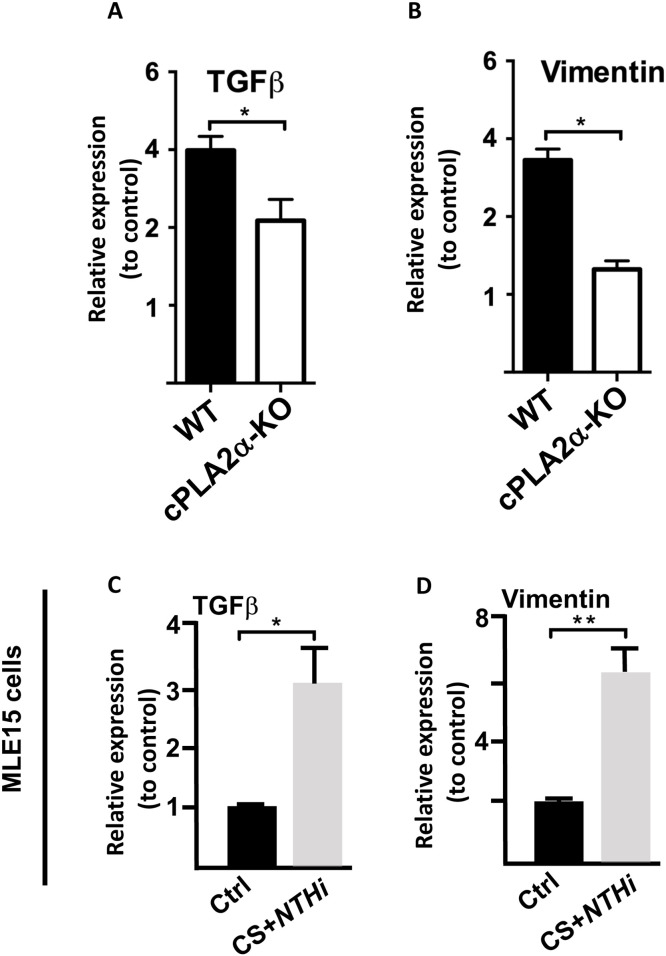
**Decreased lung TGF-β and Vimentin mRNA transcript levels in the absence of cPLA2α after chronic exposure to CS + *NTHi* infection. A,B/** Results are expressed as fold increase of transcripts in CS+NTHi-exposed lungs when compared to unexposed lungs. Data shown correspond to one pool of equal amounts of RNA from CS+*NTHi*-exposed WT or cPLA2α-KO lung tissue homogenates. Similar data were obtained in the other two pools of each genotype. Data represent the means ± SEM. *p<0.05. **Induced gene expression of TGF-β and Vimentin in cultured cells in response to CS+NTHi treatment.** Subconfluent alveolar-like epithelial cells MLE-15 cells were incubated alone or in the presence of CSE followed by *NTHi* as described above. Real-time RT-PCR was performed using equal amounts of RNA from untreated and CS+NTHi-treated cells and normalized to GAPDH. **C,D/** Shown are representative histograms corresponding to the expression data of TGF-β and Vimentin. Data represent the mean ± SEM of three values. *p<0.05, **p<0.01. Similar data were obtained in two replicate experiments. .

With respect to the observed airway epithelium thickening, we focused on TGF-β and vimentin since their implication in tissue remodeling has been documented in several reports (Hoňková et al., 2014; Rogel et al., 2011). Our data revealed a decrease in their mRNAs levels in cPLA2α-KO lungs when compared to WT lungs suggesting a role for cPLA2α in their transcription (Fig. 5A and B).

To confirm the contribution of the enzyme in the expression of these mediators, we used the alveolar-like epithelial cells, MLE-15. Similar to RAW264.7 macrophage cell line, MLE-15 cell line is a well-established murine distal airway epithelial cell line used extensively to study epithelial-pathogen interactions and cigarette smoke responses. These cells are also the first sentinel cells of contact for inhaled pathogens and major source of cPLA2α-dependent eicosanoids. Using the above optimized cell treatment conditions, we found that CSE+*NTHi* induced a significant increase in TGF-β and vimentin mRNA transcripts in MLE cells coinciding again with the detection of active cPLA2α (Fig. 5C and D). Taken together, these results corroborate our in vivo findings.

## Discussion

In the present study, we demonstrate that cytosolic phospholipase A2α (cPLA2α) plays a central role in amplifying inflammation and promoting airway epithelial thickening in a murine model of bacterial infection-mediated COPD exacerbation. Although intranasal infection with non-typeable *Haemophilus influenzae* (NTHi), a human-restricted pathogen, represents a mouse clearance model, it effectively recapitulates key inflammatory and structural features of infection-triggered COPD exacerbations.

Compared with cPLA2α-deficient mice, wild-type (WT) animals exhibited a markedly exaggerated lung response characterized by elevated inflammatory mediators and pronounced airway epithelial thickening. These in vivo findings were supported by complementary macrophage and epithelial cell culture experiments. To our knowledge, this is the first in vivo evidence implicating cPLA2α in a clinically relevant experimental model of COPD exacerbation. It represents a conceptual advance in understanding the molecular hierarchy governing COPD exacerbations.

Despite similar recruitment of macrophages, neutrophils, and lymphocytes in wild-type and cPLA₂α-deficient mice mirroring inflammatory profiles observed in human COPD (Burge and Wedzicha, 2003; Chin et al., 2005), WT lungs displayed a markedly exaggerated inflammatory mediator response and pronounced airway epithelial thickening. These findings indicate that cPLA2α does not primarily regulate immune cell influx but instead amplifies downstream inflammatory signaling and remodeling pathways once inflammation is established. Consistent with this interpretation, LTB₄ production was only transiently affected in the absence of cPLA2α during acute infection and was indistinguishable between genotypes in chronic inflammation underscoring the context-dependent role of lipid mediators in this setting. The short half-life of LTB₄ (Marleau et al., 1994; Prado et al., 2017), compensatory chemotactic mechanisms, and the involvement of alternative PLA₂ isoforms (Saiga et al., 2005; Muñoz et al., 2003; Wu et al., 2010) may collectively mask its role during prolonged inflammation. Importantly, cPLA2α deficiency did not impair bacterial clearance, indicating that its primary function in this model is inflammatory modulation rather than host defense.

Our data instead point to cPLA2α as a key upstream regulator of inflammatory amplification and airway remodeling, acting through coordinated regulation of selected mediators. Among these, OPN and MIP-2 appear to contribute to sustained inflammation. Both mediators are known to enhance pro-inflammatory cytokine production reinforcing inflammatory signaling cascades, characteristic featues of COPD exacerbations (Barkas and Kotsiou, 2023; Lee et al., 2014; Schneider et al., 2010; Driscoll, 1994; Chaochao et al., 2017). Their upregulation in WT lungs supports a role for cPLA2α in maintaining a pro-inflammatory environment.

In parallel, TGF-β and vimentin emerge as contributing drivers of airway epithelial thickening. Elevated TGF-β signaling is strongly associated with excessive extracellular matrix deposition, basement membrane expansion, and airway wall thickening in COPD, particularly during exacerbations (Takizawa et al., 2001; Königshoff et al., 2009; Colarusso et al., 2019). Vimentin, a cytoskeletal protein implicated in cellular plasticity and tissue remodeling, has been linked to epithelial structural alterations and airflow limitation in COPD patients (Ostrowska Podhorodecka et al., 2022; Chou et al., 2025). The concomitant upregulation of TGF-β and vimentin in WT but not cPLA2α-deficient lungs provides a mechanistic basis for the epithelial thickening observed in our model.

Taken together, these findings support a model in which cPLA2α might function as an upstream signaling “**switch** or hub” that promotes signal transduction via activation of transcription factors (e.g., NF-κB, AP-1, Smad, STAT3) (van Puijenbroek et al., 1999; Stuhlmeier et al., 1997) hence inflammatory amplification via OPN and MIP-2 while simultaneously driving airway epithelial thickening through TGF-β– and vimentin-dependent pathways suggesting. Crosstalk among these mediators likely sustains a feed-forward loop linking inflammation to structural airway changes, a hallmark of COPD exacerbations.

While the global cPLA2α knockout approach limits cell-specific interpretation, our data nonetheless identify cPLA2α as a critical molecular player of inflammation and remodeling. Additional complexity that may further modulate cPLA2α role arises from host-pathogen interactions. In fact, an important aspect of NTHi pathogenesis in COPD involves the outer membrane protein 1 (OmpP1), known also as Fatty Acid Degradation protein L (FadL) that has been reported to mediate uptake of AA. Recent studies showed that allelic variation in OmpP1/FadL strongly affects bacterial adaptation to the COPD lungs, where AA levels are elevated due to cPLA₂α activity (Moleres et al., 2018). In the present study, the OmpP1/FadL genotype of the NTHi 11P6 strain was not determined (and there is no public data specifically addressing “strain 11P6” and its OmpP1 expression), and its sensitivity to AA was not assessed. Consequently, the contribution of FadL allelic variation to the observed inflammatory responses remains unknown and could represent a limitation that warrants further investigation. This would clearly provide mechanistic evidence for host-pathogen interactions in COPD exacerbations and enhance our understanding by integrating host enzyme activity, lipid mediator availability, and bacterial genotype.

In conclusion, our work highlights the relative importance of cPLA2α in COPD exacerbation by orchestrating inflammatory amplification and airway epithelial thickening. Targeting cPLA2α (thus its associated signal transduction pathways), particularly through localized and selective inhibition, may therefore represent an attractive therapeutic strategy to attenuate inflammatory burden and limit cumulative airway damage without impairing host immunity during COPD exacerbations.

## Author contribution

BL performed the in vivo studies, histological studies and qPCR analyses. LM and AP performed ELISA, Gelatin zymography, and cytokine antibody microarrays. FBM and AEB performed part of the animal and culture experiments and histological and qPCR analyses. LT and AB were involved in the supervision of the project, conception of the studies, design of the experiments and their interpretation. OG, LT and AB were involved in drafting the manuscript for important intellectual content.

## Declaration of competing interest

The authors declare that they have no known competing financial interests or personal relationships that could have appeared to influence the work reported in this paper.

## Appendix Supplementary materials

Image, application 1

Image, application 2

Image, application 3

Image, application 4
