# Supplementary figures and images for: Loss of cPLA2α function attenuates inflammation and epithelial thickening in a mouse model of Haemophilus influenzae-mediated COPD exacerbation
Source: Curr Res Microb Sci. 2026 Jan 22;10:100556. doi: 10.1016/j.crmicr.2026.100556 (PMC12887665; doi:10.1016/j.crmicr.2026.100556)

Supplementary figure 1

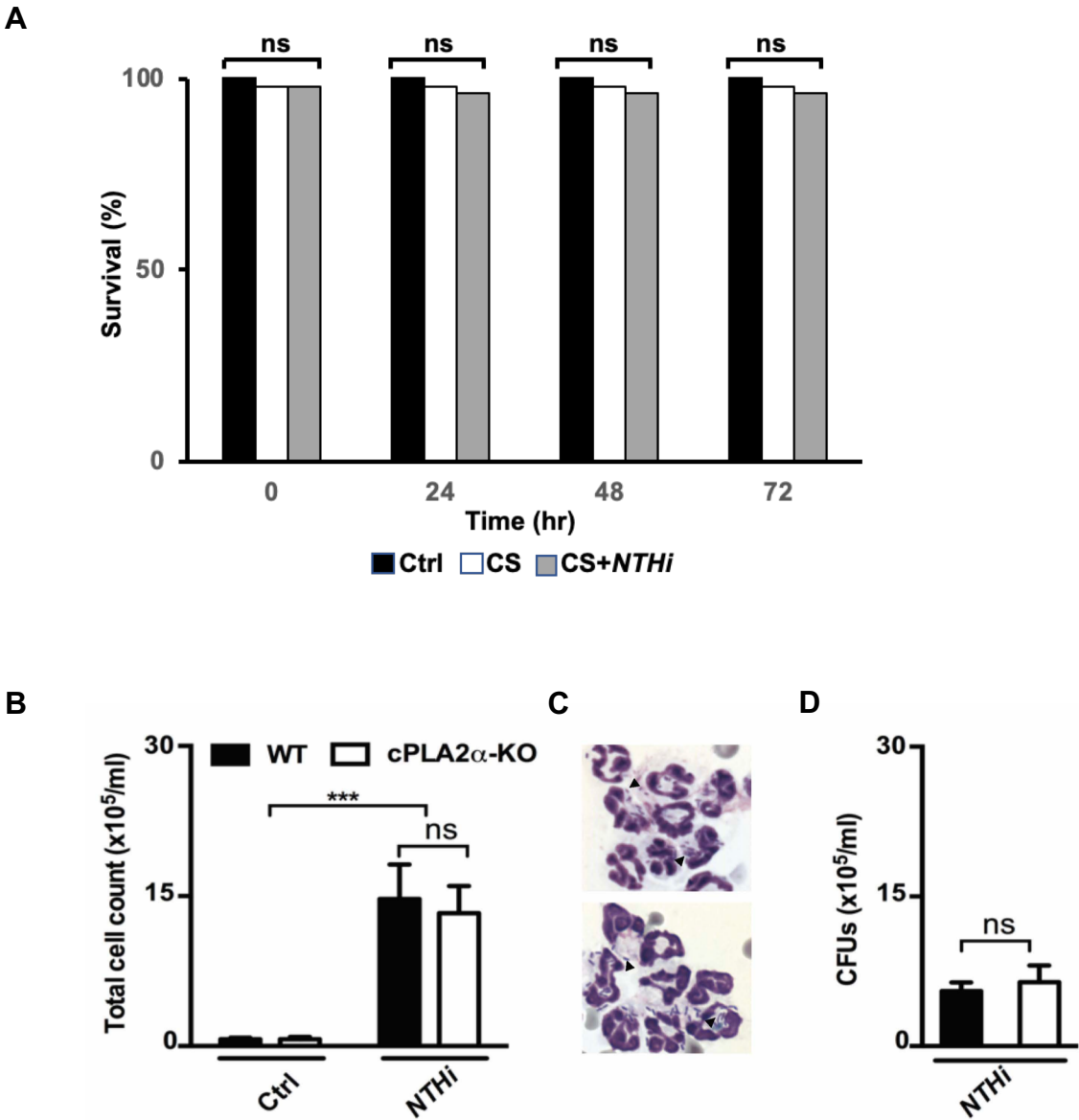

Supplement: Supplementary file 1 [file mmc1.pdf]

Supplementary figure 2

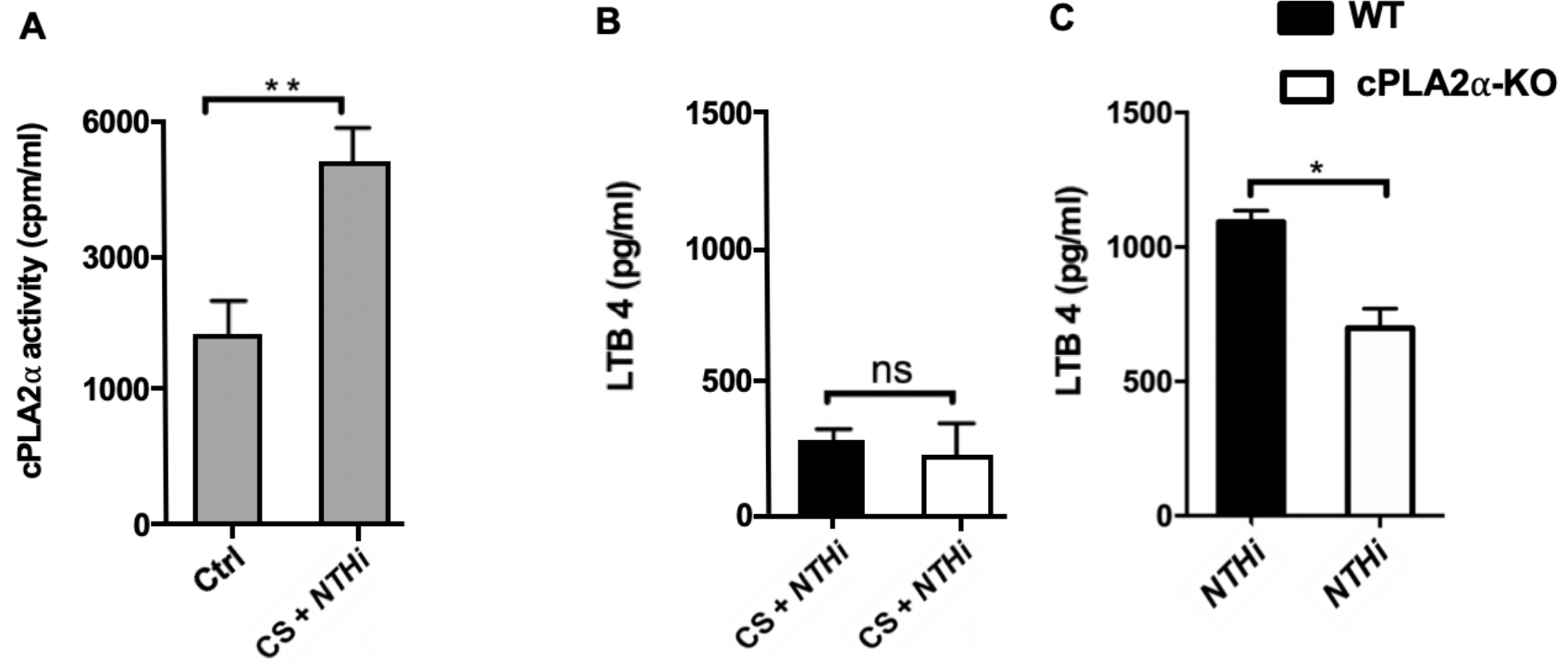

Supplement: Supplementary file 2 [file mmc2.pdf]

Supplementary figure 5

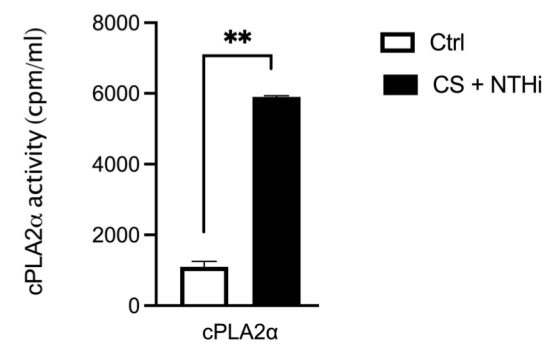

Supplement: Supplementary file 4 [file mmc4.pdf]
