# Supplementary material for: Loss of cPLA2α function attenuates inflammation and epithelial thickening in a mouse model of Haemophilus influenzae-mediated COPD exacerbation
Source: Curr Res Microb Sci. 2026 Jan 22;10:100556. doi: 10.1016/j.crmicr.2026.100556 (PMC12887665; doi:10.1016/j.crmicr.2026.100556)

Supplementary figure 3

A

Each antibody is spotted in duplicate vertically

	A	B	C	D	E	F	G	H	I	J	K	L	M	N
1<br>2	POS	POS	NEG	NEG	BLANK	Axl	BLC (CXCL13)	CD30 Ligand (TNFSF8)	CD30 (TNFRSF8)	CD40 (TNFRSF5)	CRG-2	CTACK (CCL27	CXCL16	Eotaxin-1 (CCL11)
3<br>4	Eotaxin-2 (CCL24)	Fas Ligand (TNFSF6)	Fractalkine (CX3CL1)	GCSF	GM-CSF	IFN-gamma	IGFBP-3	IGFBP-5	IGFBP-6	IL-1 alpha (IL-1 F1)	IL-1 beta (IL-1 F2)	IL-2	IL-3	IL-3 R beta
5<br>6	IL-4	IL-5	IL-6	IL-9	IL-10	IL-12 p40/p70	IL-12 p70	IL-13	IL-17A	KC (CXCL1)	Leptin R	Leptin	LIX	L-Selectin (CD62L)
7<br>8	Ltn (XCL1)	MCP-1 (CCL2)	MCP-5	M-CSF	MIG (CXCL9)	MIP-1 alpha (CCL3)	MIP-1 gamma	MIP-2	MIP-3 beta (CCL19)	MIP-3 alpha (CCL20)	PF-4 (CXCL4)	P-Selectin	RANTES (CCL5)	SCF
9<br>10	SDF-1 alpha	TARC (CCL17)	I-309 (TCA-3/CCL1)	TECK (CCL25)	TIMP-1	TNF alpha	TNF RI (TNFRSF1A)	TNF RII (TNFRSF1B)	TPO	VCAM-1 (CD106)	VEGF-A	BLANK	BLANK	POS

Each antibody is spotted in duplicate vertically

	A	B	C	D	E	F	G	H	I	J	K	L
1<br>2	POS	POS	NEG	NEG	BLANK	bFGF	CD26 (DPPIV)	Dtk	E-Selectin	Fc gamma RIIB	Flt-3 Ligand	GITR (TNFRSF18)
3<br>4	HGFR	ICAM-1 (CD54)	IGFBP-2	IGF-1	IGF-2	IL-15	IL-17 RB	IL-7	I-TAC (CXCL11)	Lungkine (CXCL15)	MDC (CCL22)	MMP-2
5<br>6	MMP-3	OPN (SPP1)	OPG (TNFRSF11B)	Pro-MMP-9	Resistin	Shh-N	TCK-1 (CXCL7)	TIMP-2	TRANCE (TNFSF11)	TROY (TNFRSF19)	TSLP	VEGFR1
7<br>8	VEGFR2	VEGFR3	VEGF-D	BLANK	BLANK	BLANK	BLANK	BLANK	BLANK	BLANK	BLANK	POS

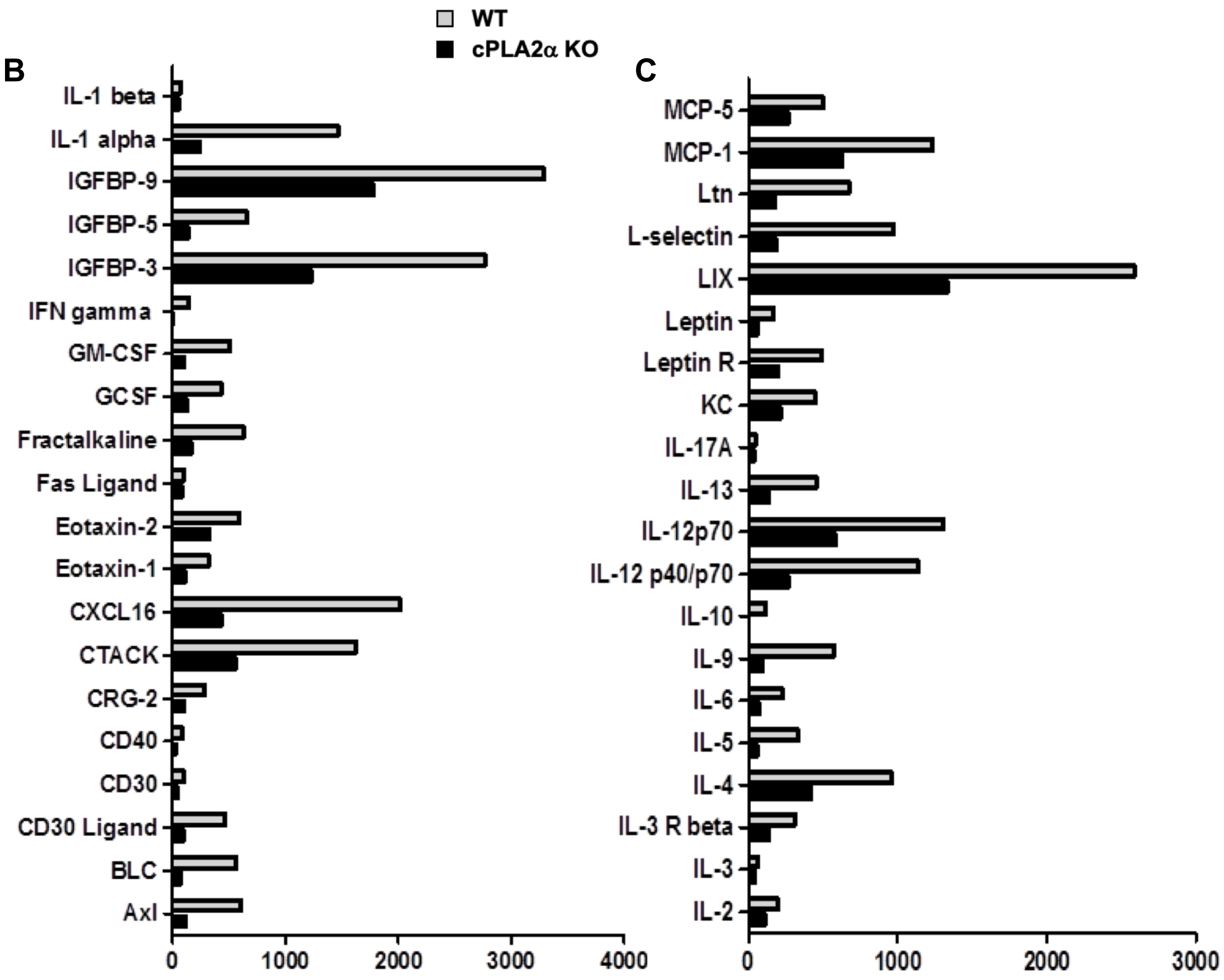

Supplementary figure 4

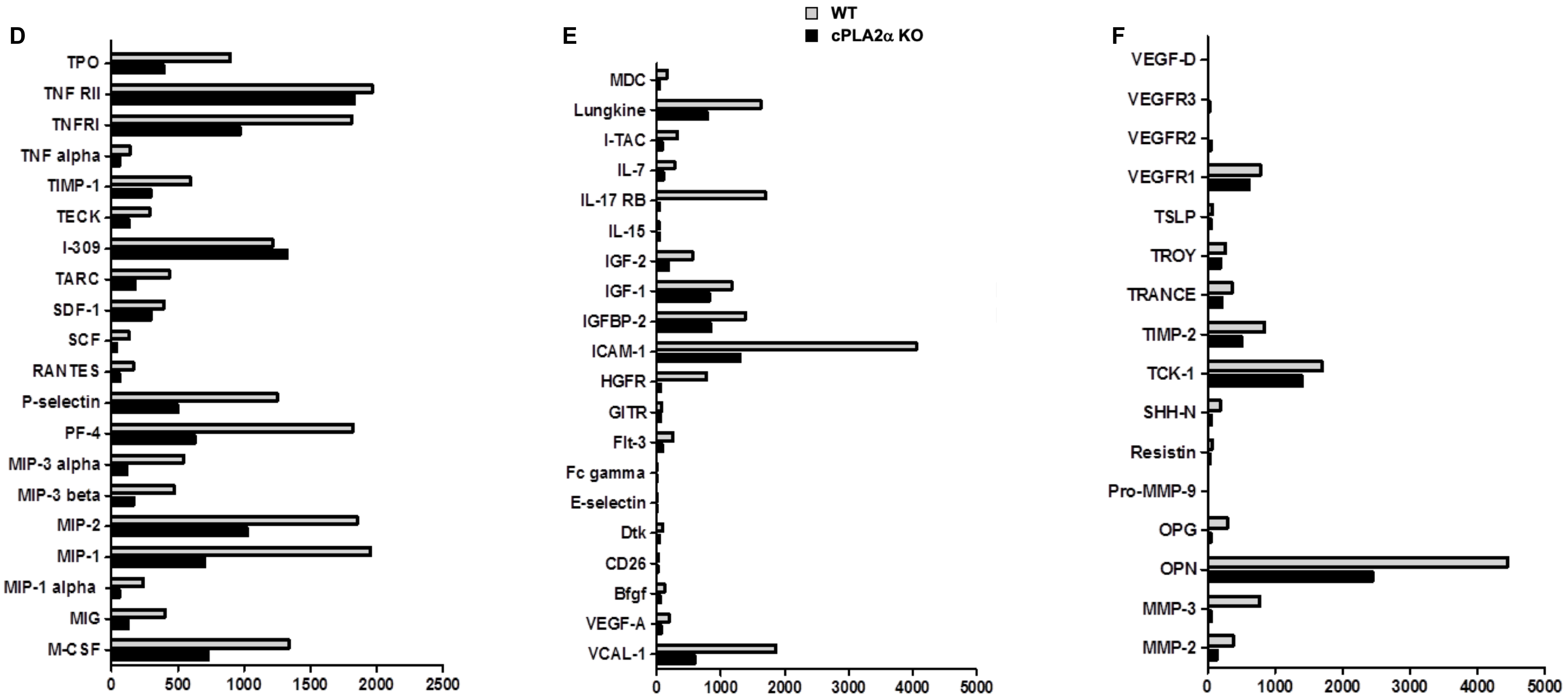

Supplement: Supplementary file 3 [file mmc3.pdf]
